# Rapid antibiotic susceptibility testing of bacteria from patients’ blood via assaying bacterial metabolic response with surface-enhanced Raman spectroscopy

**DOI:** 10.1038/s41598-020-68855-w

**Published:** 2020-07-27

**Authors:** Yin-Yi Han, Yi-Chun Lin, Wei-Chih Cheng, Yu-Tzu Lin, Lee-Jene Teng, Juen-Kai Wang, Yuh-Lin Wang

**Affiliations:** 1 Department of Anesthesia, National Taiwan University Hospital, Taipei, Taiwan; 2 Department of Traumatology, National Taiwan University Hospital, Taipei, Taiwan; 3 Institute of Atomic and Molecular Sciences, Academia Sinica, Taipei, Taiwan; 4 Department of Clinical Laboratory Sciences and Medical Biotechnology, National Taiwan University, Taipei, Taiwan; 5 Center for Condensed Matter Sciences, National Taiwan University, Taipei, Taiwan; 6 Center of Atomic Initiative for New Materials, National Taiwan University, Taipei, Taiwan; 7 Department of Physics, National Taiwan University, Taipei, Taiwan; 8 Present Address: Department of Medical Laboratory Science and Biotechnology, China Medical University, Taichung, Taiwan

**Keywords:** Microbiology techniques, Raman spectroscopy, Nanoparticles, Nanophotonics and plasmonics, Sensors, Nanosensors, Raman spectroscopy, Raman spectroscopy

## Abstract

Blood stream infection is one of the major public health issues characterized with high cost and high mortality. Timely effective antibiotics usage to control infection is crucial for patients’ survival. The standard microbiological diagnosis of infection however can last days. The delay in accurate antibiotic therapy would lead to not only poor clinical outcomes, but also to a rise in antibiotic resistance due to widespread use of empirical broad-spectrum antibiotics. An important measure to tackle this problem is fast determination of bacterial antibiotic susceptibility to optimize antibiotic treatment. We show that a protocol based on surface-enhanced Raman spectroscopy can obtain consistent antibiotic susceptibility test results from clinical blood-culture samples within four hours. The characteristic spectral signatures of the obtained spectra of *Staphylococcus aureus* and *Escherichia coli*—prototypic Gram-positive and Gram-negative bacteria—became prominent after an effective pretreatment procedure removed strong interferences from blood constituents. Using them as the biomarkers of bacterial metabolic responses to antibiotics, the protocol reported the susceptibility profiles of tested drugs against these two bacteria acquired from patients’ blood with high specificity, sensitivity and speed.

## Introduction

Blood stream infection (BSI)—defined as the presence of viable bacteria in blood (*i.e.*, bacteremia) documented by a positive blood culture result^1^—arouses morbidity and mortality worldwide^2^. It is the primary cause of sepsis, which is a major public health concern with particular high medical cost^3,4^. Although the number of BSI incidence bears demographic difference, it shows increasing trend over time^5,6^ and therefore remains an urgent medical challenge. Timely administration of appropriate antibiotic therapy is critical to avoiding its progression. A retrospective analysis study based on a large dataset showed that there was a linear increase in the risk of mortality for each hour delay in antibiotic administration^7^, which was confirmed by a recent study^8^. However, owing to lack of timely microbiological evidence, antibiotics are usually forced to start empirically, rather than precisely upon specific target(s)^9^. Improper antibiotic selection was not uncommon and the mortality markedly increased^10^. Moreover, the initial empirical use of broad-spectrum antibiotics^11^, although prudent, inevitably contributes to the increasing prevalence of drug-resistant bacteria in hospitals. According to the first annual report by Review on Antimicrobial Resistance, the continued rise in resistance by 2050 would lead to 10 million people dying per year and a reduction of 2–3.5% in gross domestic product^12^.

Blood culture is the first step in the current standard methods used to diagnose bacteremia. At least 12 h are needed to allow bacterial multiplication providing a rich source of live bacteria for downstream bacteria identification and antibiotic susceptibility testing (AST). Clinical and Laboratory Standards Institute (CLSI) regularly revises the standard AST protocols that demand additional overnight incubation to determine the minimal inhibitory concentration (MIC) of tested antibiotic for ultimate determination of bacterial resistogram^13^. The final results usually take 3–4 days in total (or longer for fastidious organisms) to obtain the final AST report even under the aids of automated instruments. In contrast to the rapid deterioration of BSI course, the labor-intensive cultivation procedure seems particularly delaying, resulting in complications of various severe conditions, including mortality.

A number of new techniques have been devised to meet the need for rapid microbiological diagnosis^14–17^. Some of them were tested in antimicrobial stewardship intervention programs^18^. Verigene Gram-negative and Gram-positive blood culture microarray-based assays were recently demonstrated to reduce the detection time of resistant bacteria^19^. FilmArray (bioMérieux, Inc, Hazelwood, Mo) Blood Culture Identification Panel by BioFire Diagnostics is a multiplex PCR system that can identify three antibiotic-resistant genes and promises reduction in AST time^20^. Semi-quantitative matrix-assisted laser desorption ionization-time of flight mass spectroscopy (MALDI-TOF MS) identified antibiotic-treated strains by the intensity reduction in their mass spectra^21, 22^. An up-to-date review of the application of such technique to antimicrobial resistance is given by Ovlaño and Bou^23^. Vitek 2 (bioMérieux, Inc, Hazelwood, Mo), an automated version of standard microdilution method, detects resistant strains with low error rates^24–26^. Phoenix system (Becton Dickinson Diagnostic Systems, Sparks, MD) determines antibiotic resistance by fluorescence monitoring of discharged CO_2_ level of viable bacteria under antibiotic treatment^27^. We note that a recent study evaluated the performance of the Accelerate Pheno System (Accelerate Diagnostics, USA) in identification and AST of Gram-negative bacteria from blood-culture samples^28^. The system identifies bacteria based on gel electrofiltration and fluorescence in situ hybridization, and analyzes bacterial growth rates and determines MIC values based on automated microscopy. These modern AST methods however seldom satisfy all the requirements of an ideal microbiological diagnostic method: specificity, sensitivity, rapidity, and reliability. Therefore, development of new rapid AST methods remains an active area of research.

Goodacre’s group^29^ and our group^30^ exploited surface-enhanced Raman spectroscopy (SERS) to reveal the vibrational fingerprints of bacteria on metallic nanostructures. We identified simple SERS markers specific to Gram-negative and Gram-positive bacteria of reference strains and of clinical isolates from culture for several generations, and used them to perform antibiotic susceptibility testing and to determine minimal inhibitory concentration—dubbed SERS-AST method^31^. Another group illustrated SERS detection of bacteria spiked in whole-blood samples^32^. The robust vibrational spectroscopic signatures of bacteria acquired by SERS were found to originate primarily from the metabolites of purine degradation rather than cell wall structures: adenine, hypoxanthine, xanthine, guanine, uric acid, and adenosine monophosphate^33,34^. The findings provide the biochemical basis for the development of SERS as a rapid bacterial diagnostic method.

With recent advancements in reliable substrates, integrated SERS technology and understanding about the bacterial metabolomics, SERS has high potential to supplement or even replace some of the existing time-consuming AST methods for patients with infection in blood and may help alleviate the growing drug resistance. However, the high complexity of blood stream infections makes it extremely challenging to fulfill the potential, not only because of the diversity of clinically occurred bacteria which may behave differently from reference strains, but also because of the spectral interferences created by the complex constituents in clinical blood samples. Among them, hemoglobin is particularly troublesome because it is known to exhibit strong intrinsic SERS spectrum^35^. In order to advance the SERS-based microbiological diagnostic method from simplistic scenario on laboratory benches to complex real world on hospital bedsides, a comprehensive and applicable protocol needs to be established. In this proof-of-principle study, we have developed a process for treating clinical bacteremic samples and demonstrated its integration with our previously established SERS-based method. A rapid AST working on bacteria from patients’ blood via assaying bacterial metabolic response with SERS was achieved, representing a major step of implementing the SERS-AST method in clinical microbiology.

## Materials and methods

### Study design

This study aimed to set up a SERS-based microbial diagnostic protocol focusing on antibiotic susceptibility testing for patients with blood stream infections. The protocol consists of three major sections: (1) preparation of clinical blood-culture samples, (2) antibiotics treatment, and (3) SERS measurement. While the latter two refer to the prototypic flow set on reference strains and pure clinical isolates in our previous SERS-AST study^31^, the first one was newly developed to meet the requisite for advancing the built technology to more complicated clinical bacteremia setting. The study was conducted in National Taiwan University Hospital (NTUH)—a tertiary care hospital in Taiwan. Patient identification and confidentiality were maintained.

### Patient selection

*Staphylococcus aureus* (*S. aureus*) and *Escherichia coli* (*E. coli*) were chosen as the test targets of Gram-positive bacteria and Gram-negative bacteria, respectively, in this study to demonstrate the performance of the SERS-AST method on clinical positive blood-culture samples acquired from patients with blood stream infections. The high occurrence rate of both bacterial species in BSI patients and the accessibility of SERS measurements in our previous study on reference strains and pure clinical isolates were the two main reasons for this designation. To screen the eligible target species, daily preliminary reports of all positive blood-culture bottles yielded by the automated Vitek 2 system (Advanced Expert System software, version 7.0; bioMérieux) in the Laboratory department of NTUH were checked. The blood-cultures that grew either *S. aureus* or *E. coli* were potentially eligible once the following host factors were excluded: pregnancy, age under 18 years and polymicrobial infections. The former two exclusion criteria are set only to avoid the vulnerable groups for whom more complicated processes are needed for either ethical board review or study inclusion. After the informed consent was obtained, 5 ml of the mixture of blood and culture medium was aspirated from the positive culture bottles (Bactec Plus Aerobic/F or Anaerobic/F culture bottles) and transferred to serum separator tubes. During the study period, a total of 75 clinical strains of *S. aureus* (*n* = 32), and *E. coli* (*n* = 43) were enrolled. The relevant hospitalization data were also recorded.

### Pretreatment of clinical blood-culture samples

**Figure 1:**
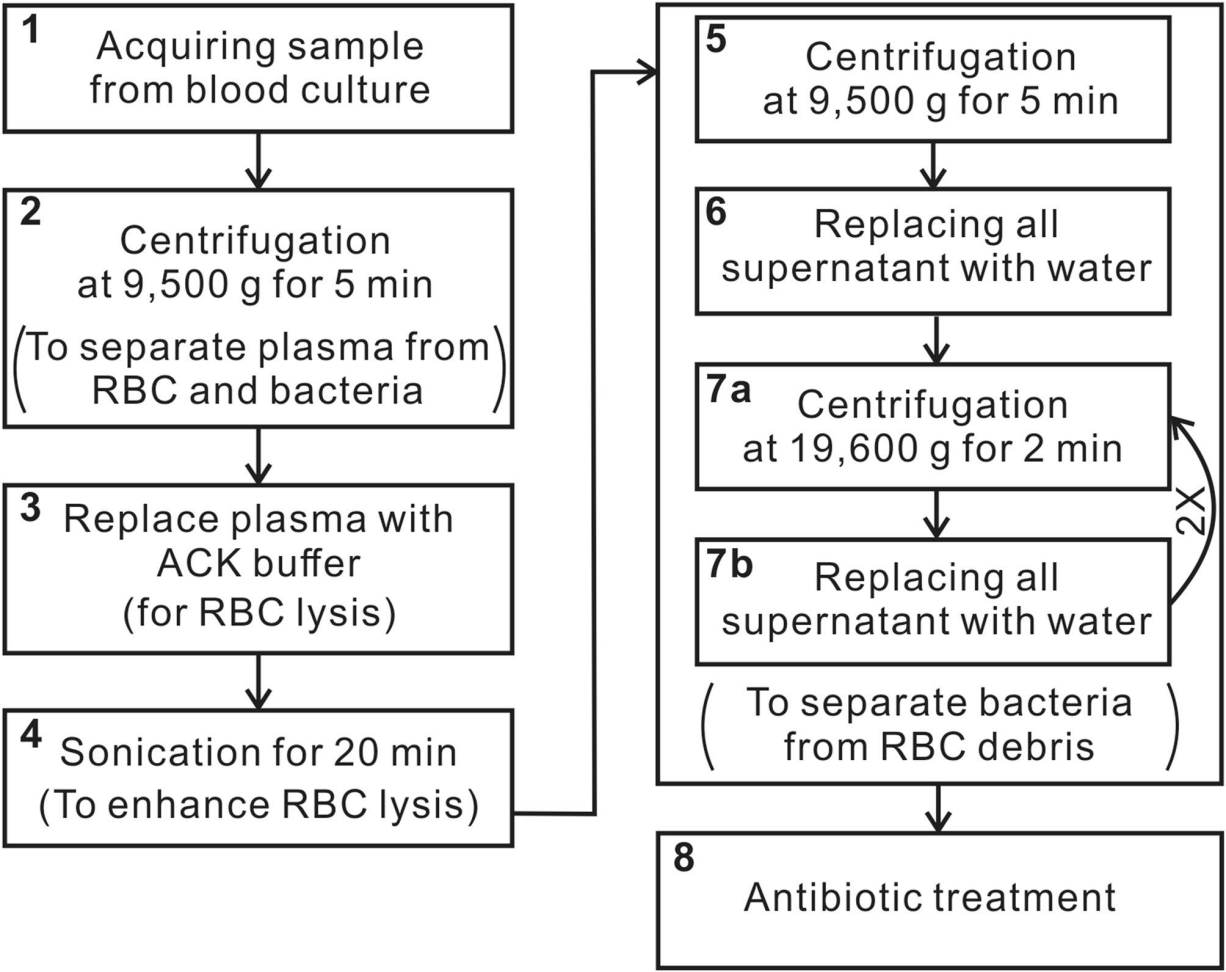
Pretreatment procedure of blood-culture samples. Selective lysis process was optimized to separate blood components from bacteria, while being non-nondestructive to the microorganisms. The whole procedure is composed of eight steps. Step 7a and 7b are repeated twice (indicated with 2 ×).

Selective lysis process was optimized to separate blood components from bacteria, while being nondestructive to the microorganisms. The blood-culture samples were first treated by the stepwise procedure shown in Fig. 1 to extract the bacteria. After the acquirement of the positive blood-culture samples (Step 1), 5 ml of the sample placed in a 15-ml tube then underwent centrifugation at 9,500×*g* for 5 minutes (Step 2), leaving the bacteria precipitated on the bottom of the tube. The plasma above the bacterial pellet was then replaced by ACK (Ammonium-Chloride-Potassium) buffer (Step 3) to lyse red blood cells. Ultrasonication of the mixture was then carried out by a sonicator (SK3310HP, KUDOS) at 53 kHz, 180 W and 30 °C for 20 min. (Step 4) to disrupt cell membranes and release cellular contents, such as hemoglobin from the red blood cell (RBC)—so called sonoporation^36,37^—more thoroughly, while avoiding bacteria inactivation^38–40^. After the resultant sample tube underwent the same centrifugation procedure (Step 5), the supernatant—containing hemoglobin, cellular fragments and other low-density components, *etc*.—was replaced with sterile water (Step 6). The sample was then washed twice by the same procedure adopted from our previous work^31^, which includes centrifugation at 19,600×*g* for 2 min. (Step 7a) and replacement of the 4.8 ml supernatant part by the same amount of sterile water (Step 7b), to remove the buffer and remnant constituents of blood cells. The viable bacteria left in the sediment were referred to “blood-culture isolates.” A proportion of the isolates was used for tests following the previously developed SERS-AST protocol^31^ that includes (1) adjustment of bacterial concentration, (2) inoculation in solutions of antibiotics, (3) SERS measurement, and (4) determination of MIC. The rest of isolates were stored at -80°C for ultimate evaluation with standard agar dilution method^41,42^.

### Antibiotic treatment

**Figure 2:**
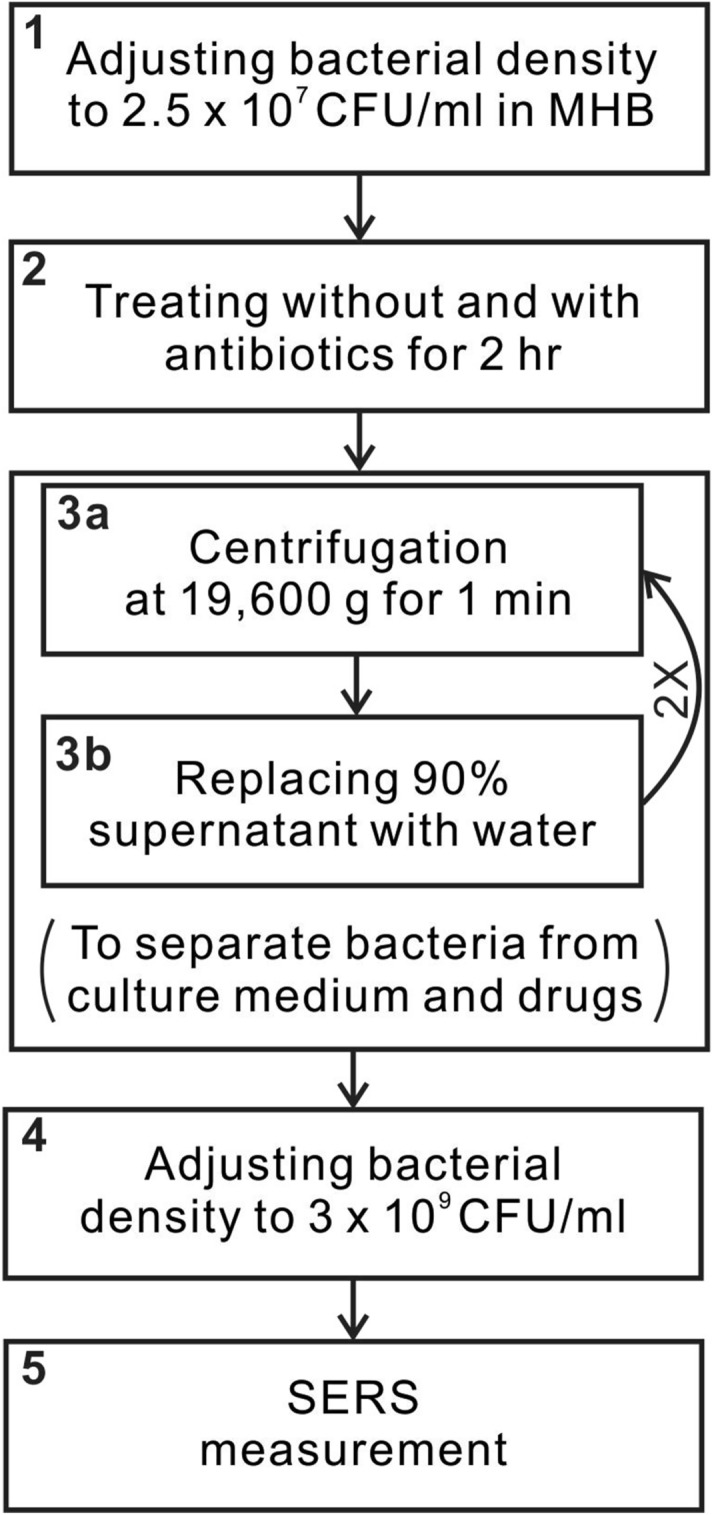
Procedure of antibiotic treatment. MHB stands for Mueller Hinton broth. Step 3 is repeated twice (indicated with 2 ×).

Following the general rules of clinical antibiotic usage^43^, we selected oxacillin (OXA) and cefotaxime (CTX) for *S. aureus* and *E. coli*, respectively. Both lyophilized antibiotics (Merck KGaA) were dissolved separately in 2 ml regenerating solutions to yield concentration of 0.25 mg/ml. The prepared drug stock solutions were stored at − 20 °C for up to 7 days following the instruction from the manufacture. Figure 2 shows the four-step antibiotic treatment procedure before SERS measurement. The blood-culture isolates mixed in Mueller Hinton (MH) broth were first adjusted to a density of 2.5 × 10^7^ CFU/ml (Step 1)—characterized by its optical density (OD) at 600 nm with an ELISA reader (Multi-Mode Microplate Readers, Molecular Devices)—before antibiotic treatment. According to the standard antibiotic susceptibility profile provided by CLSI^13^, *S. aureus* is considered susceptible to OXA for its minimum inhibitory concentration (MIC) below 2 μg/ml and resistant for above 4 μg/ml, while *E. coli* is considered susceptible to CTX for its MIC below 1 μg/ml, intermediate for 2 μg/ml, and resistant for higher than or equal to 4 μg/ml. Five cell culture tubes with 2-ml bacterial suspension were prepared to contain five drug concentrations (0.5, 1, 2 and 4 μg/ml) leaving one without drug as control. The one without zero drug concentration served as the control. All the tubes were then incubated in the thermostat (OSI-500R, TKS) at 37 °C for 2 h, followed by SERS measurement.

### SERS substrate

The substrates used in SERS measurement were silver-nanoparticle array embedded in nanochannels of anodic aluminum oxide. The fabrication procedure^31,44^ is briefly described here. A glass slide was coated with a 100-nm aluminum film by sputtering. The aluminum-coated glass slide was then anodized in sulfuric acid under a bias voltage to create a two-dimensional hexagonally packed array of nanochannels with an average inter-channel spacing of about 50 nm. The nanochannels were further etched chemically to yield an average channel diameter of 45 nm, resulting in a mean gap of 5 nm between adjacent nanochannels. Silver were then grown into the nanochannels via electrochemical plating to produce an array of silver nanoparticles with an average length of 60 nm. Individual SERS-active substrates were then cleaned by rinsing with deionized water, followed by being vacuum-sealed in plastic bags for storage. To minimize surface contamination, each substrate was freshly used immediately after the bag was unsealed. The quality of each SERS-active substrate was evaluated with adenine test (1 × 10^−4^ M)^44^ before applying for bacterial assay. Background, signal strength, and signal-to-background ratio were examined at four spots separated by >1 cm on the substrate. Only substrates with variation of these quantities within 15% were adopted for the subsequent SERS-AST assay. The rejection rate of the substrates in this work is less than 10%. The spots used for uniformity testing on the substrate were avoided in the subsequent SERS measurements of bacteria.

### SERS measurement

After 2-h incubation with antibiotics, all the samples underwent the wash procedure (Step 7 in Fig. 1) twice to remove the drug as well as the culture medium. It is assumed the recovery yield is identical to the control for the 4 antibiotics samples. The bacterial pellet of the control sample acquired after the centrifugation in the wash procedure was suspended in deionized water so that the bacterial density was 3 × 10^9^ CFU/ml according to its optical density at 600 nm in deionized water, while the pellets of the other four antibiotic-treated samples were suspended in the same amount of deionized water used for adjusting the bacterial density of the control sample. One microliter of each bacterial sample was drawn with a pipette and placed on the SERS-active substrate, forming a circular spot with a diameter of ~1.5 mm. Four spots of the bacterial samples treated with antibiotics of four different concentrations were aligned in a horizontal row and separated by ~1 mm on the SERS-active substrate, while four spots of the control sample aligned in the other horizontal row were separated vertically from the antibiotic-treated sample spot row by a gap of ~1 mm, forming a 4 × 2 spot array on the SERS substrate. To minimize the potential measurement error owing to the would-be non-uniformity of the SERS-active substrate, the control sample spots were placed nearby the antibiotic-treated sample ones. The SERS substrate with the placed bacterial sample spots were then dried on a hot plate by keeping the substrate at 52–55 °C for 15 min. to dry the sample droplets quickly to facilitate the following SERS measurement.

All SERS measurements were performed with a Raman microscope built with a spectrometer (HE 633, Horiba) plus a thermoelectric-cooled charge-coupled device (CCD), a Raman probe (Superhead, Horiba), and an upright optical microscope (B × 61WI, Olympus). A He–Ne later emitting at 632.8 nm served as the excitation source. The laser beam was delivered via an excitation optical fiber to the Raman probe in which its residual plasma lines was removed by a laser-line filter. The beam was then focused by a 20 × objective lens to the bacterial sample spots on the substrate surface. The typical laser irradiation power density at the substrate surface was about 1 × 10^5^ mW/cm^2^. The scattered radiation was collected backward by the same objective lens, passed through a long-pass filter inside the Raman probe, and eventually was delivered by an emission optical fiber to the spectrometer plus thermoelectric-cooled CCD for spectral recording. The resultant spectral resolution and error are 20 and 3 cm^−1^, respectively. The integration times of the Raman spectra were set to be 1 second. For each sample spot on the SERS substrate, Raman measurements were performed at 8–10 randomly chosen laser-focused positions within the sample spot. Special attention was paid to prevent any other laser-induced effects such that the spectral patterns thus obtained were stable during signal accumulation and the signal strength was linearly proportional to the irradiating laser power and the integration time. After the rarely occurred spectra that exhibited dissimilar spectral patterns or excessively large background were removed, the remaining spectra underwent removal of baseline and cosmic-ray peaks with sensitive nonlinear iterative peak clipping algorithm and then averaging, described in our previous work^31^. Specifically, two criteria were adopted to remove the outliers in the measured SERS spectra: (1) the spectrum containing anomalous peaks that are absent in the mean SERS spectrum of *E. coli* or *S. aureus*; (2) the spectrum bearing a signal-to-background ratio of less than 2. The first case occurs when some contaminations are present on the laser focal spot of the SERS substrate, while the latter case occurs when the laser focal spot of the substrate accidently exhibit low enhancement. The specific bacterial SERS spectrum of each sample was presented and evaluated. As a final note, since the light-wave propagation from the laser, to the Raman probe combined with the optical microscope, and from Raman probe to the spectrometer plus CCD were through optical fibers, no delicate optical alignment was needed and the instrument maintenance was simple. This unique propensity makes the Raman system most suitably for operation in hospitals.

### Extracting biomarker signals

Following the protocol in our previous SERS study on reference strains and pure clinical isolates^31^, the spectral peaks located at 730 and 724 cm^−1^ were identified and adopted as the SERS biomarkers of *S. aureus* and *E. coli*, respectively, in this study. The signal ratios of the intensities of the SERS biomarker of the four antibiotic-treated samples to that of the untreated one were calculated. That is, for *S. aureus*, the signal ratio at 730 cm^−1^ was derived and labeled as $r_{730} = S_{730}^{{\text{D}}} /S_{730}^{0}$, where $S_{730}^{{\text{D}}}$ and $S_{730}^{0}$ are the peak signals at 730 cm^−1^ of the bacterial samples with and without the antibiotic challenge, respectively. Similar data processing was applied to the case of *E. coli*, yielding the signal ratio at 724 cm^−1^—$r_{724} = S_{724}^{{\text{D}}} /S_{724}^{0}$, where $S_{724}^{{\text{D}}}$ and $S_{724}^{0}$ are the corresponding signals of the peak at 724 cm^−1^ for the samples of *E. coli*.

### Antibiotic susceptibility tests with agar dilution method

Standard AST with agar dilution method^41,42^ was performed for all the blood-culture isolates, following the instruction of CLSI guideline^13^. Designated antibiotics (OXA or CTX) of different concentrations, ranging from 0.125 to 256 μg/ml, were mixed in agar plates. Bacterial suspension of each blood-culture isolate of 10^8^ CFU/ml—corresponding to optical opacity equivalent to 0.5 McFarland turbidity standard—was prepared in normal saline using the colonies grown from subculture. The bacterial inoculum of 10^4^ CFU per spot was delivered to each plate using a Multiple Inoculator (Steers Replicator). Blank agar plates without antibiotics were included in each bacterium-drug series as the control. After incubation at 37 °C for 16–18 h, the growth of each bacterial inoculum on plates of sequential drug concentrations were assessed for MIC determination.

### Receiver operating characteristic analysis

**Figure 3:**
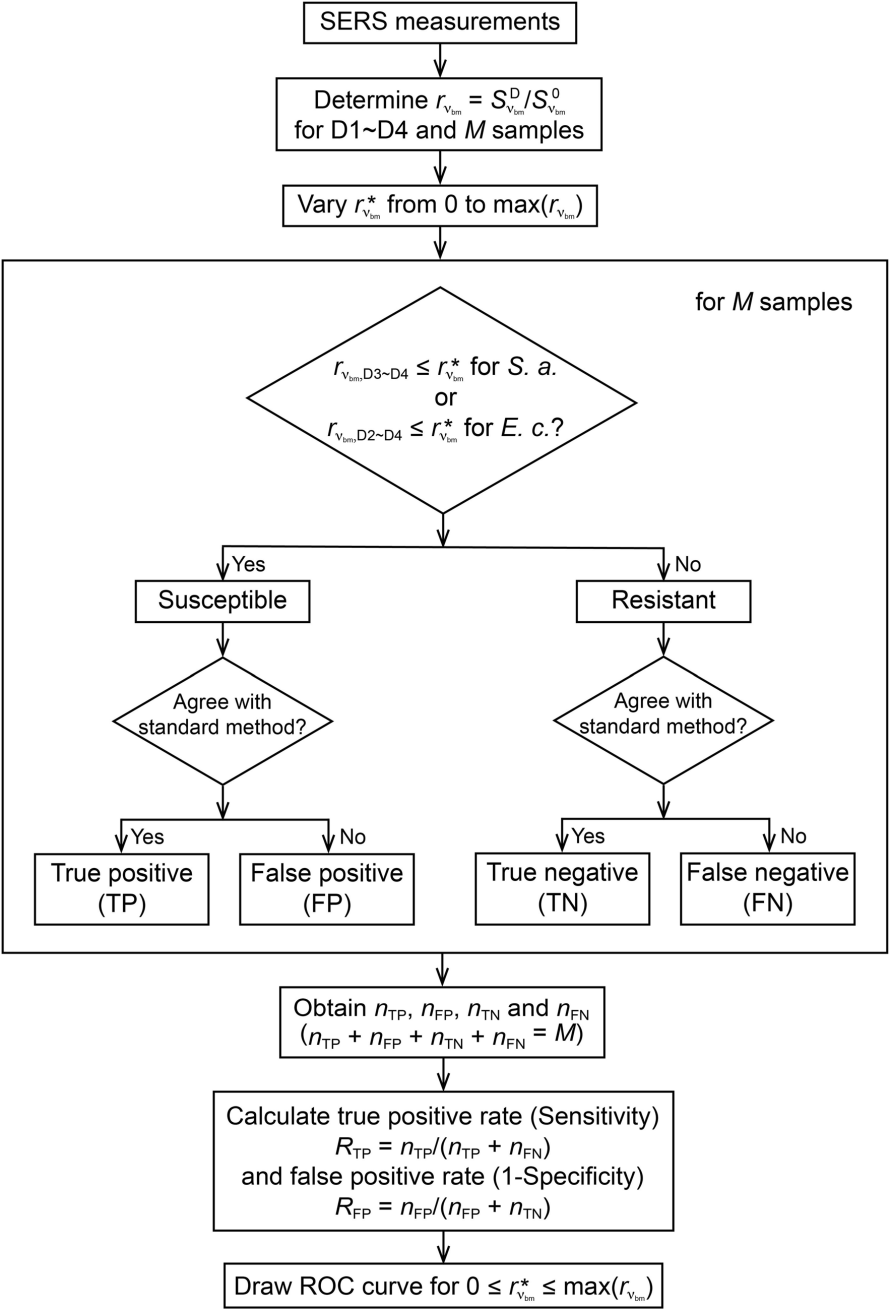
Flow chart of receiver operating characteristic (ROC) analysis. $S_{{\nu_{bm} }}^{D}$ and $S_{{\nu_{bm} }}^{0}$ are the SERS signals at the Raman shift of the biomarker peak,$\nu_{{{\text{bm}}}}$, with and without antibiotic treatment, respectively. $\nu_{{{\text{bm}}}}$ is 730 cm^−1^ for *S. aureus* and is 724 cm^−1^ for *E. coli*. $r_{{\nu_{{{\text{bm}}}} }}$ is the ratio between $S_{{\nu_{{{\text{bm}}}} }}^{{\text{D}}}$ and $S_{{\nu_{{{\text{bm}}}} }}^{0}$. D1 indicates the lowest drug concentration, D2 indicates the second lowest drug concentration, and so on. $r_{{\nu_{{{\text{bm}}}} }}^{*}$ is the cutoff signal ratio. $r_{\nu_{{\text{bm}}}, {\text{Dj}}}$ is the signal ratio at $\nu_{{{\text{bm}}}}$ of the bacterial sample treated with the antibiotic of concentration D*j*. *E. c.* and *S. a.* are the abbreviations of *E. coli* and *S. aureus*, respectively. $n_{{{\text{TP}}}}$, $n_{{{\text{FP}}}}$, $n_{{{\text{TN}}}}$ and $n_{{{\text{FN}}}}$ are the numbers of true positive, false positive, true negative and false negative cases, respectively. *M* is the total number of samples. $\max \left( {r_{{\nu_{{{\text{bm}}}} }} } \right)$ is the maximal measured $r_{{\nu_{{{\text{bm}}}} }}$ obtained from the *M* samples.

Receiver operating characteristic (ROC) analysis was adopted to assess the optimal cutoffs of the two signal ratios of the SERS biomarkers to determine their respective drug MICs and susceptibilities. The signal ratios of the SERS biomarker ($r_{730}$ for *S. aureus* and $r_{724}$ for *E. coli*) of the four drug concentrations were calculated for each blood-culture isolate. The antibiotic susceptibility profile of each blood-culture isolate was determined according to the CLSI guideline for AST: For *S. aureus*, if the signal ratios of the two highest drug concentrations (2 and 4 μg/ml) were both smaller than or equal to the cutoff signal ratios ($r_{{730,{\text{D}}3\sim {\text{D}}4}} \le r_{730}^{*}$), that specific isolate was considered susceptible to the drug; for *E. coli*, if the signal ratios of the three highest drug concentrations (1, 2 and 4 μg/ml) were all smaller than or equal to the cutoff signal ratios ($r_{{724,{\text{D}}2\sim {\text{D}}4}} \le r_{724}^{*}$), that was considered susceptible to the drug. D1 represents the drug treatment with the lowest concentration, D2 represents that with the second lowest concentration, and so on. On the other hand, if the signal ratios corresponding to the three lower drug concentrations (0.5, 1 and 2 μg/ml) were larger than the cutoff signal ratio and the one corresponding to the highest concentration (4 μg/ml) was larger than or comparable to the cutoff signal ratio ($r_{{\nu_{{{\text{bm}}}} ,{\text{D}}1\sim {\text{D}}3}} > r_{{\nu_{{{\text{bm}}}} }}^{*}$ and $r_{{\nu_{{{\text{bm}}}} ,{\text{D}}4}} \ge r_{{\nu_{{{\text{bm}}}} }}^{*}$, where $\nu_{{{\text{bm}}}}$ is the Raman shift of the biomarker), the specific isolate was considered resistant to the drug. The obtained susceptibility pattern of that specific isolate was then compared with that obtained with the standard agar dilution method. If the two AST profiles agree, the SERS-AST result obtained is either true positive or true negative; on the other hand, if the two profiles are inconsistent, the SERS-AST result is either false positive or false negative. By varying the cutoff signal ratio for *S. aureus* (*E. coli*) from 0 to the maximal measured value obtained from all the samples of *S. aureus* (*E. coli*), true and false positive rates ($R_{{{\text{TP}}}}$ and $R_{{{\text{FP}}}}$, respectively) were subsequently determined: $R_{{{\text{TP}}}} = n_{{{\text{TP}}}} /\left( {n_{{{\text{TP}}}} + n_{{{\text{FN}}}} } \right)$ and $R_{{{\text{FP}}}} = n_{{{\text{FP}}}} /\left( {n_{{{\text{FP}}}} + n_{{{\text{TN}}}} } \right)$, where $n_{{{\text{TP}}}}$, $n_{{{\text{FP}}}}$, $n_{{{\text{TN}}}}$ and $n_{{{\text{FN}}}}$ are the numbers of true positive, false positive, true negative and false negative cases, respectively. For a specific cutoff signal ratio, the resultant $R_{{{\text{TP}}}}$ (Sensitivity) and $R_{{{\text{FP}}}}$ (1-Specificity) thus give a data point in the ROC graph. The details of the ROC analysis are depicted in Fig. 3. The area under the curve (AUC) was considered as an effective assessment of the accuracy for the SERS-AST method. The optimal cutoff ratios of *S. aureus* and *E. coli* were the ones which maximized their respective Youden’s indices (*J*):

1 $$J = {\text{Sensitivity}} + \left( {{\text{Specificity}} - 1} \right).$$

The MIC defined by the SERS-AST test was the lowest drug concentration where the signal ratio of the SERS biomarker drops below the optimal cutoff signal ratio. The ultimate susceptible profile of each blood-culture isolate to the tested drug (susceptible, intermediate or resistant) was determined by comparing the MIC results with the international performance standards for AST set by CLSI^13^. The final outcome was categorized as: agreement, very major error (false susceptible), major error (false resistant), and minor error (susceptible/resistant versus intermediate susceptibility). The percentages of very major error, major error, and minor error cases (corresponding to $\eta_{{{\text{VME}}}}$, $\eta_{{{\text{ME}}}}$ and $\eta_{{{\text{mE}}}}$, respectively) were calculated according to the ISO 20776-2 standard: $\eta_{{{\text{VME}}}} = n_{{{\text{FR}}}} /n_{{\text{R}}}$, $\eta_{{{\text{ME}}}} = n_{{{\text{FS}}}} /n_{{\text{S}}}$ and $\eta_{{{\text{mE}}}} = n_{{\text{F}}} /M$, where $n_{{{\text{FR}}}}$ is the numbers of resistant isolates that were not categorized as resistant, $n_{{{\text{FS}}}}$ is the numbers of susceptible isolates that were not categorized as susceptible, $n_{{\text{R}}}$ is the total number of resistant isolates tested, $n_{{\text{S}}}$ is the total number of susceptible isolates tested, $n_{{\text{F}}} = n_{{{\text{FR}}}} + n_{{{\text{FS}}}}$, and $M = n_{{\text{R}}} + n_{{\text{S}}}$. Note that we consider the ‘intermediate’ isolates to be resistant ones in this study.

## Results

### SERS detection of positive blood-culture isolates

**Figure 4:**
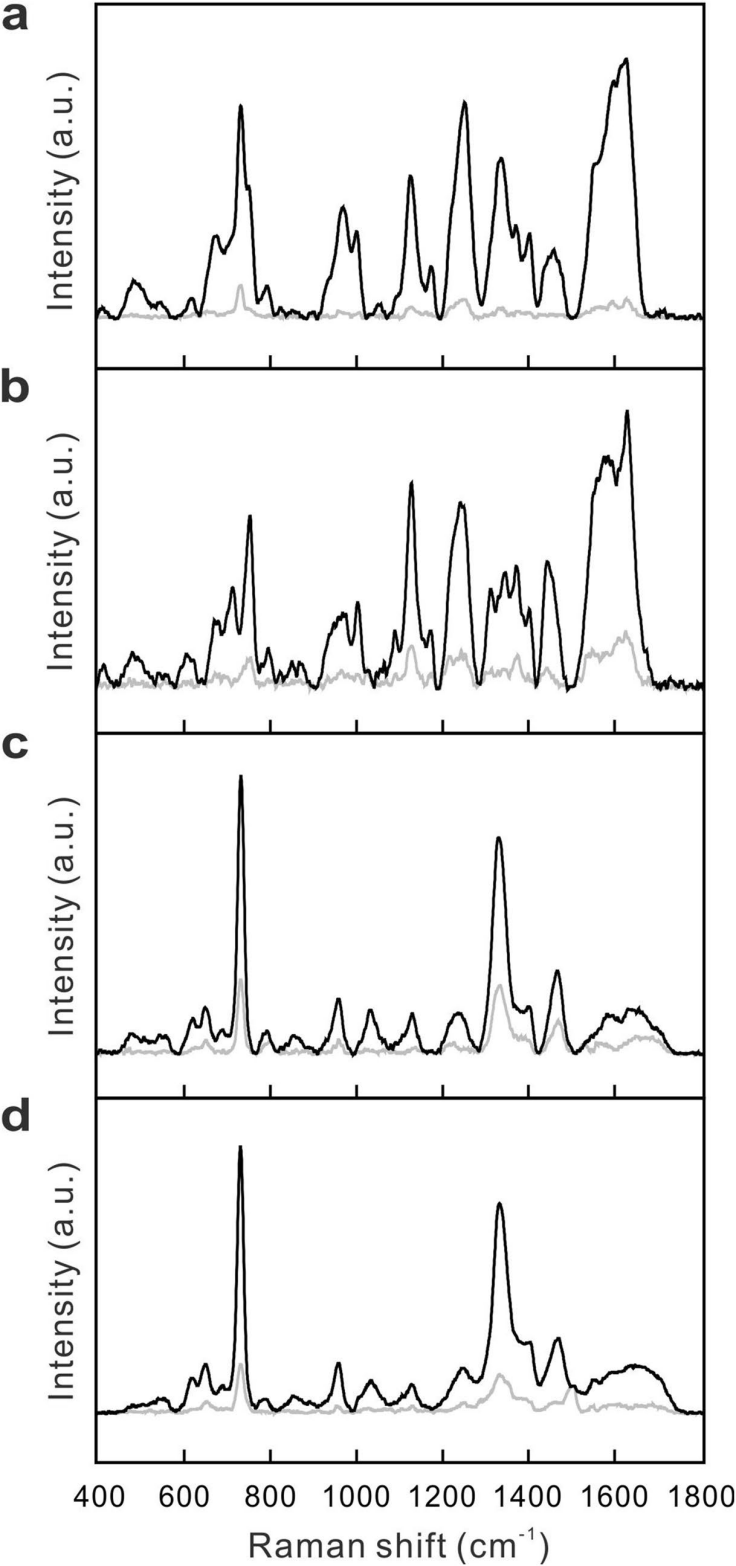
SERS spectra without sample pretreatment. SERS spectra of (**a**) goat blood spiked with *S. aureus* (1 × 10^9^ CFU/ml) without ACK treatment and sonication, (**b**) hemoglobin, (**c**) reference strain of *S. aureus*, and (**d**) goat blood spiked with *S. aureus* with ACK treatment and sonication. Black curves represent the mean SERS spectra, while gray curves represent their corresponding standard deviation.

**Figure 5:**
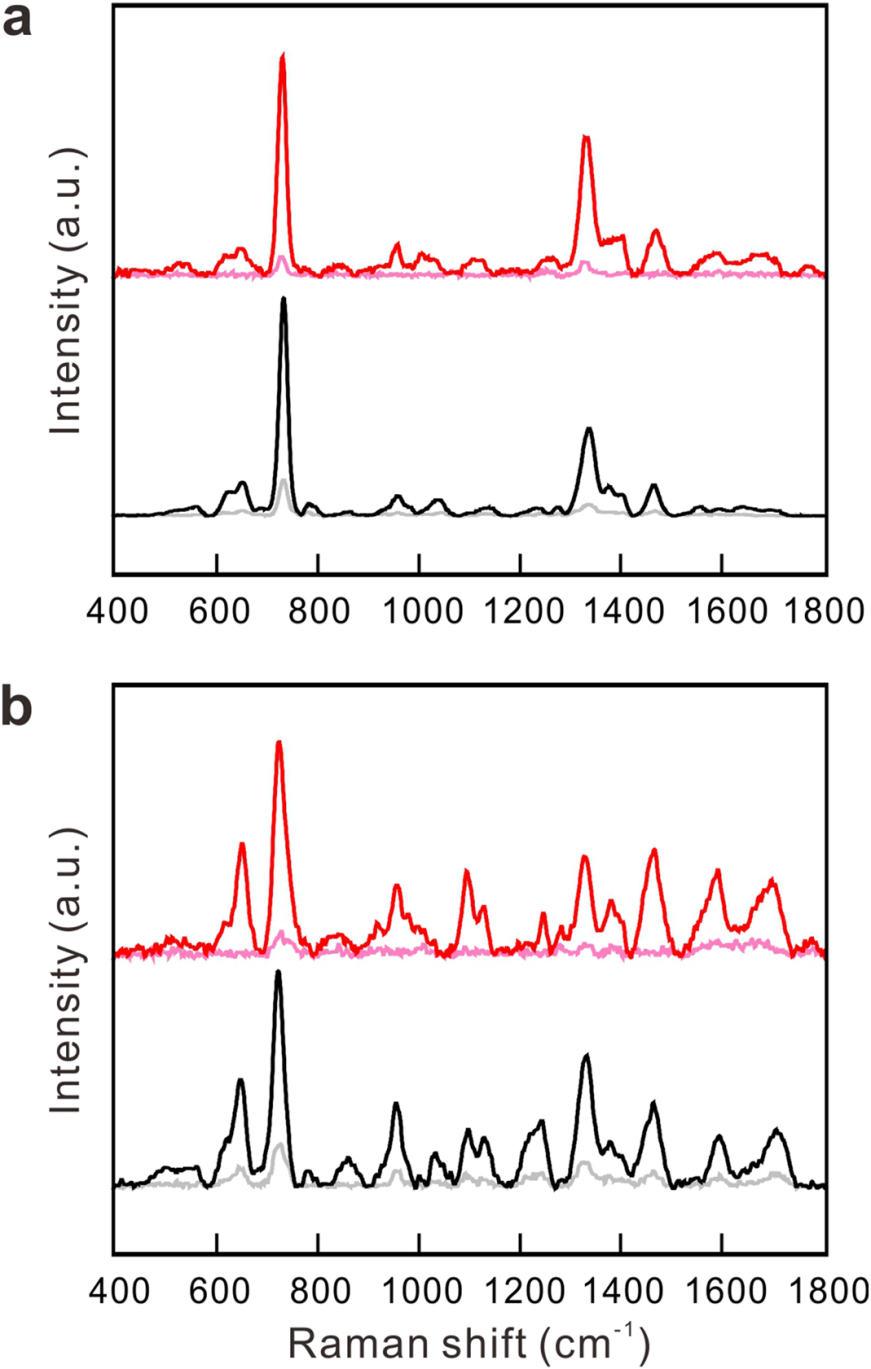
Removal of interfering SERS signal with sample pretreatment. (**a**) is for *S. aureus* and (**b**) is for *E. coli.* Red and black curves represent the mean SERS spectra of blood-culture isolates and reference strains, respectively, while light red and grey curves represent their corresponding standard deviation.

The major challenge to conduct SERS measurement of bacteria in blood-culture samples is to remove all the spectral interferences from the other constituents in cultured blood samples—including culture media, blood cells, fragmented DNA, RNA, proteins, fat, and medications, etc.—before the SERS measurement. To emulate the blood-culture samples, goat blood was spiked with *S. aureus* of 1 × 10^9^ CFU/ml. Figure 4a exemplifies the SERS spectrum of the emulated sample treated with the sample preparation procedure in Fig. 1 except the Steps 2 ~ 4 (namely, without the ACK buffer treatment and sonication). The portrayed spectrum is almost identical to that of hemoglobin, shown in Fig. 4b, and is in great contrast to that of reference *S. aureus* strain, shown in Fig. 4c. Namely, the SERS spectrum of the bacteria-spiked blood sample is dominated by hemoglobin. With the ACK buffer treatment and sonication, the resultant SERS spectrum, shown in Fig. 4d, bears great resemblance to that of the reference strain shown in Fig. 4c, indicating that hemoglobin in the emulated sample was effectively removed. Accordingly, two inferences can be made from these results: (1) the ACK buffer treatment and sonication have lysed the cell membrane of all red blood cells, thus releasing hemoglobin in the treated sample; (2) the released hemoglobin as well as other constituents are effectively removed by the washing procedure. The result establishes a firm foundation to prepare the clinical blood sample for the subsequent procedure of the SERS-AST method. Figure 5 shows the SERS spectra of the two positive blood-culture samples of *S. aureus* and *E. coli* treated by the same sample preparation procedure, as well as those of their corresponding reference strains. The SERS spectra of the two blood-culture isolates are almost indistinguishable from those of their corresponding reference bacteria. The results in both cases demonstrate that the 730-cm^−1^ peak in the SERS spectrum of the extracted *S. aureus* and the 724-cm^−1^ peak in that of the extracted *E. coli* from their blood-culture isolates can serve as the respective biomarkers in the following AST for all blood-culture samples, just as what had been done in our previous work on reference strains and clinical isolates^31^. The determined SERS biomarkers of all the blood-culture isolates (32 strains of *S. aureus* and 43 strains of *E. coli*) agreed with the classification results obtained from Gram staining test. There was no misidentification in all the 75 cases.

### Antibiotic susceptibility testing

**Figure 6:**
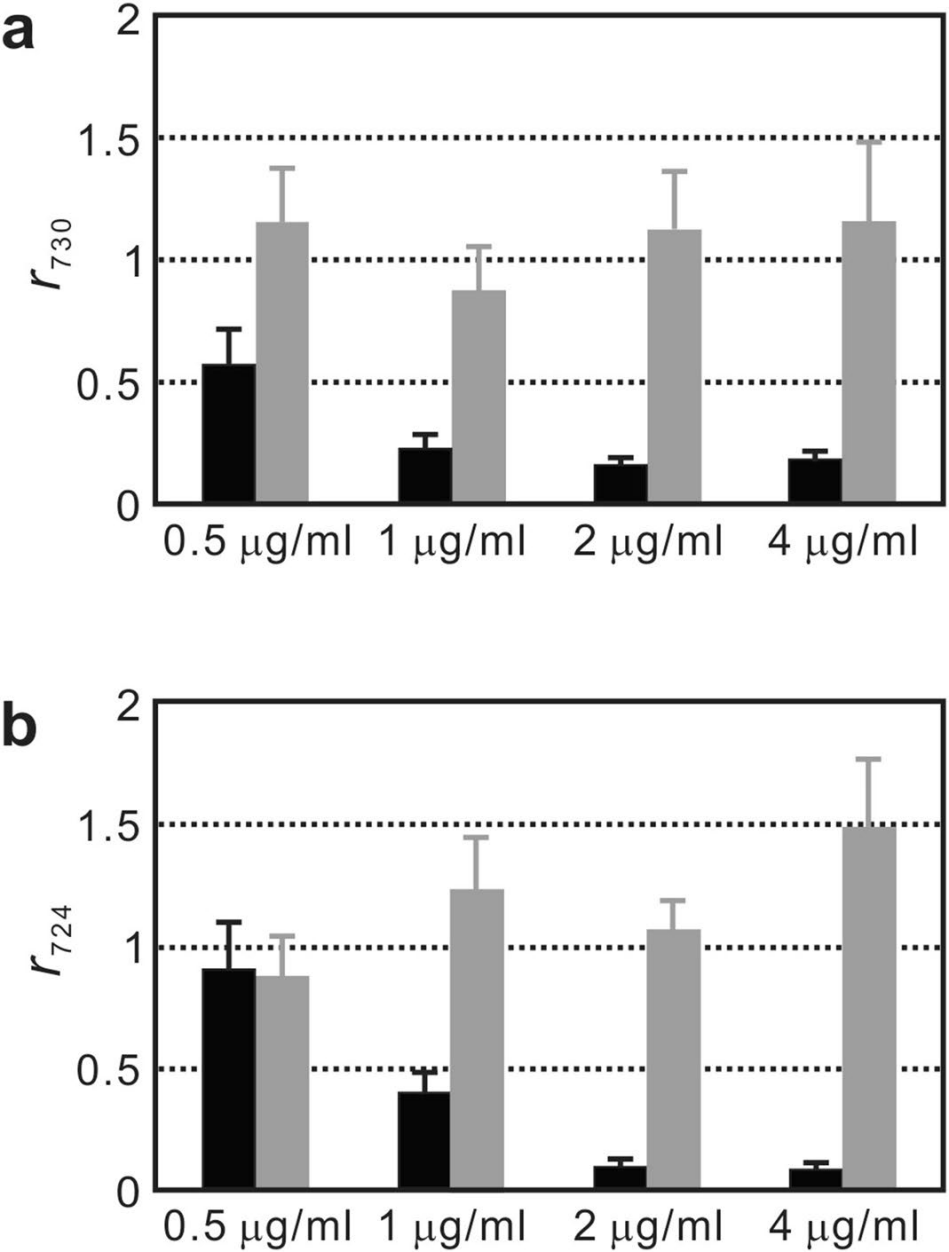
Antimicrobial responses of SERS biomarker signal. (**a**) Signal ratios of the SERS peak at 730-cm^−1^ ($r_{730}$) of a susceptible and a resistant blood-culture isolate of *S. aureus* are plotted against oxacillin concentration; (**b**) signal ratios of the SERS peak at 724-cm^−1^ ($r_{724}$) of a susceptible and a resistant blood-culture isolate of *E. coli* are plotted against cefotaxime concentration. Black columns represent the susceptible isolates, while gray columns stand for the resistant isolates.

The signal ratios of the bacterial SERS biomarker at 730 cm^−1^ for *S. aureus* ($r_{730}$) and that at 724 cm^−1^ for *E. coli* ($r_{724}$) were used to indicate their respective antimicrobial responses. Figure 6a shows the signal ratios of representative blood-isolates of susceptible and resistant *S. aureus* under the 2-h. treatment of four different oxacillin concentrations, while Fig. 6b shows those of susceptible and resistant *E. coli* under the 2-h. treatment of four different cefotaxime concentrations. The SERS spectra that were used to deduce the data shown in Fig. 6 are shown in Supporting Information (Fig. S1). For the drug concentrations higher than 0.5 μg/ml, the mean values of $r_{730}$ of the susceptible *S. aureus* isolate after the 2-h. drug treatment are below 0.5. In contrast, for all the drug concentrations, the mean $r_{730}$ values of the resistant *S. aureus* isolate are above 0.5. The similar behavior was observed in the case of the blood-culture isolates of the susceptible and resistant *E. coli* treated with four cefotaxime concentrations. The results obtained from the blood-culture isolates of *S. aureus* and *E. coli* agree respectively with the ones of our previous work on corresponding reference strains and clinical isolates^31^.

**Figure 7:**
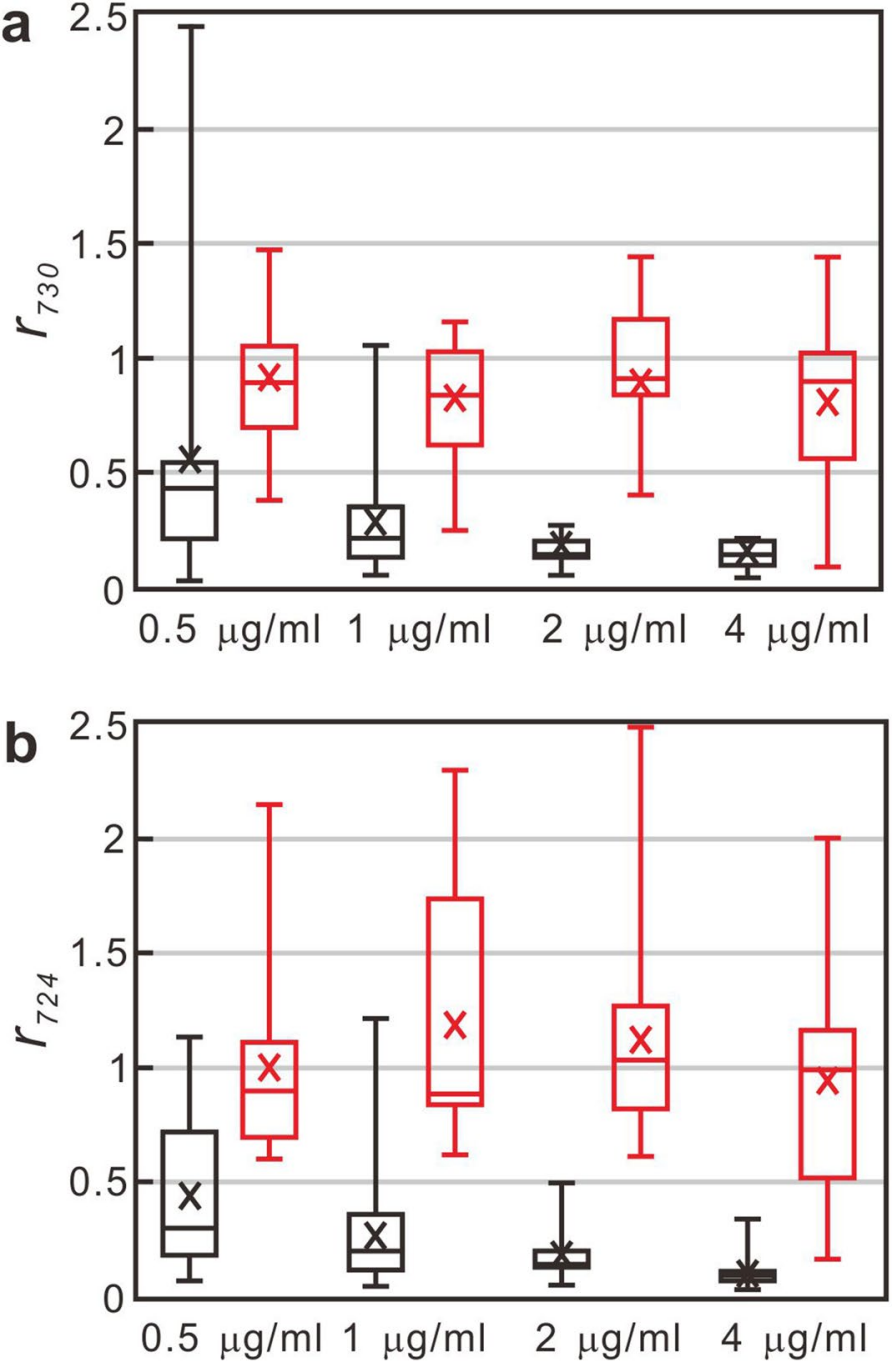
Box-and-whisker plots of SERS biomarker signal versus antibiotic concentration. (**a**) is for *S. aureus* while (**b**) is for *E. coli*. The boxes represent the 25th to 75th percentiles, with the 50th percentiles shown within the boxes. The 10th and 90th percentiles are shown as capped bars. The crosshatches inside the boxes are their respective mean values. Red and black boxes represent the data of resistant and susceptible blood-culture isolates, respectively.

Figure 6 further displays the box-and-whisker plots of $r_{730}$ and $r_{724}$ of all blood-culture isolates at the four antibiotics concentrations. The mean values of $r_{730}$ of susceptible *S. aureus* isolates decreases monotonically from 0.6 under the treatment of 0.5 μm/ml oxacillin to 0.2 under the treatment of 4 μm/ml oxacillin, while those of resistant *S. aureus* isolates remain fairly constant at around 0.8 at all the four drug concentrations (Fig. 7a). Please also note that the spread of $r_{730}$ of the susceptible isolates decreases approximately with the drug concentration, while it varies little for the resistant isolates. As a consequence, when the drug concentration is above 2 μg/ml, the $r_{730}$ distributions of the susceptible and resistant isolates are well separated. Similar character is observed in the case of *E. coli*, as shown in Fig. 7b: (1) the mean $r_{724}$ values of susceptible isolates decrease with the cefotaxime concentration and are below 0.5 for all the drug concentrations; (2) the mean $r_{724}$ values of resistant isolates are above 0.5 and vary little with all the cefotaxime concentrations. Consequently, the breakpoint concentration that discriminates the susceptible and resistant isolates can be defined for susceptibility categorization in CLSI guideline for AST (2 μg/ml oxacillin for *S. aureus* and 1 μg/ml cefotaxime for *E. coli*). Unexpected elevation of the signal ratio of the SERS biomarker is noted in some resistant isolates. Table S1 in the Supporting Information shows all the obtained signal ratios at different drug concentrations of all the positive blood-culture isolates. These aberrant data would engender some uncertainty in the choice of the cutoff signal ratio of the SERS biomarker for differentiating susceptible and resistant blood-culture isolates via ROC curve analysis and thus would influence the resultant successful rate of AST.

**Figure 8:**
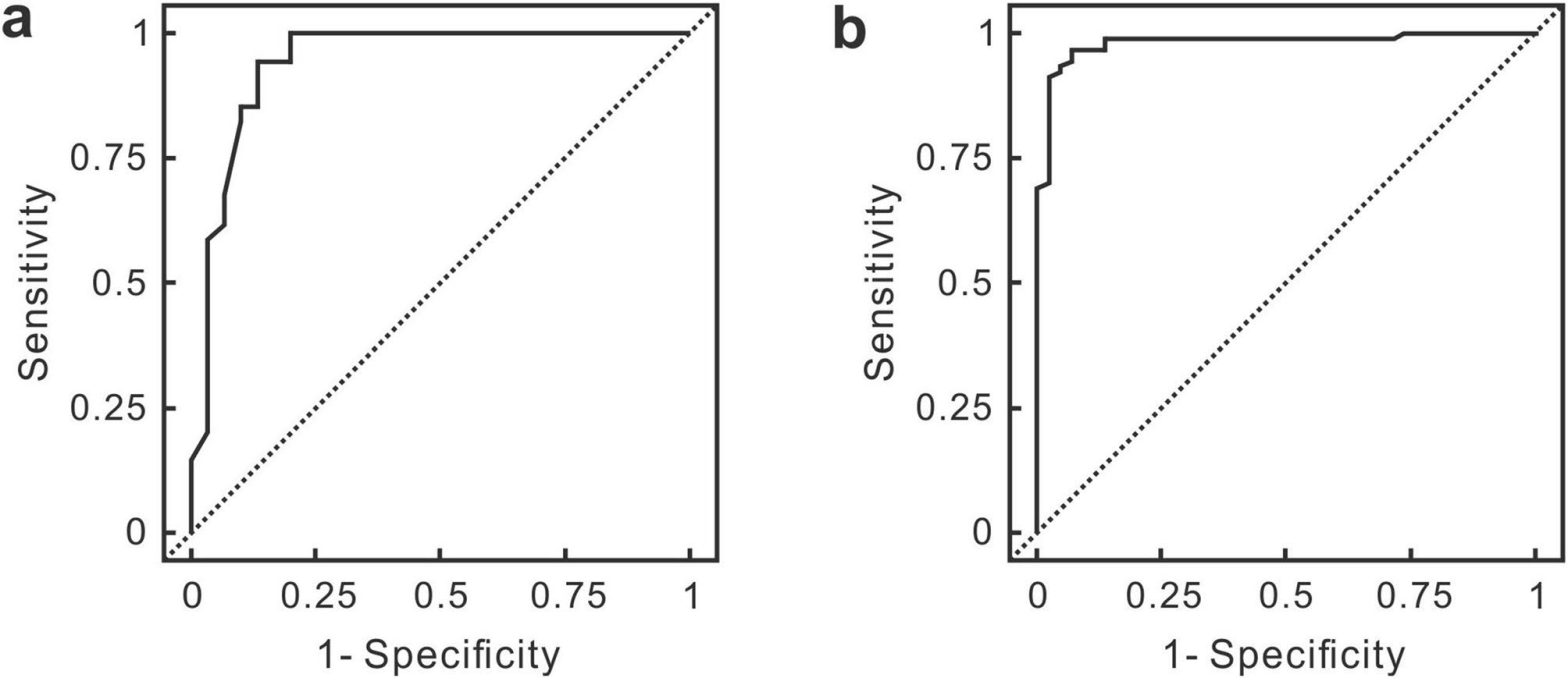
ROC analysis of SERS-AST results. (**a**) is for *S. aureus* and (**b**) is *E. coli*.

ROC analysis was applied to evaluate the performance of the SERS-AST method in detecting the antibiotic susceptibility of the blood-culture isolates. Conventional agar dilution method was used as the comparative standard in the analysis. The area under the ROC curve near 1 (0.942 for *S. aureus* and 0.981 for *E. coli*) signifies superior specificity and sensitivity of the SERS biomarker for AST working on blood-culture isolates of either *S. aureus* or *E. coli* (Fig. 8). For both *S. aureus* and *E. coli*, the obtained optimal cutoff signal ratio of the biomarker signal to discriminate the susceptible and resistant SERS patterns was yielded by maximizing the Youden’s index. Their results are presented in Table 1: The optimal cut-off signal ratio value for *S. aureus* is 0.27 (Sensitivity: 0.94; Specificity: 0.87) and that for *E. coli* is 0.50 (Sensitivity: 0.97; Specificity: 0.93).

**Table 1:** Cutoff signal ratio, sensitivity and specificity.

Bacteria	r*	Sensitivity	Specificity
*S. aureus*	0.27	0.94	0.87
*E. coli*	0.50	0.97	0.93

The MIC values of the blood-culture isolates of *S. aureus* and *E. coli* were determined with the derived optimal cutoff signal ratios of their respective biomarkers and then the bacterial susceptibility profiles of each blood-culture isolates to the selected antibiotics were categorized according to the international performance standards for AST set by CLSI^13^. The results of MIC and AST yielded by SERS were compared with those by standard agar dilution method, shown in Tables 2 and 3, respectively. The concordant rate of MIC results is 60% overall—46.9% for *S. aureus*, and 74.4% for *E. coli*, respectively. Nevertheless, the mismatches within one or two-dilution discordance do not cause substantial influence on susceptibility categorization. As to the final result, among the 32 blood-culture isolates of *S. aureus*, there were only one very major error and one major error, yielding an agreement rate of 93.8%; among the 43 blood-culture isolates of *E. coli*, there were only three minor errors, giving an agreement rate of 93.0%. Together the total agreement rate is 93.3%.

**Table 2:** Concordant statistics of minimum inhibitory concentrations.

MIC results	Bacterial strain	
	*S. aureus*	*E. coli*
Concordant	15	32
One-dilution discordant	11	1
Two-dilution discordant	6	10

Minimum inhibitory concentrations (MICs) of oxacillin for blood isolates of *S. aureus* and of cefotaxim for blood isolates of *E. coli* determined by SERS-AST method compared with those determined by standard agar dilution method.

**Table 3:** Antimicrobial susceptibility testing.

Bacteria/antibiotics	Total	Resistogram			Agreement	No. of errors		
		S	I	R		VME	ME	mE
*S. aureus*/OXA	32	17	0	15	30 (93.8%)	1 (6.7%)	1 (5.9%)	0
*E. coli*/CTX	43	30	0	13	40 (93.0%)	0	0	3 (7.0%)
Total	75	47	0	28	70 (93.3%)	1 (3.6%)	1 (2.1%)	3 (4%)

The numbers of blood-culture *S. aureus* and *E. coli* isolates under SERS-AST are 32 and 43, respectively. The antibiotic used for *S. aureus* is oxacillin (OXA), while that for *E. coli* is cefotaxime (CTX). S, I and R stand for susceptible, intermediate and resistant, respectively. The percentages of categorized agreement and very major error (VME), major error (ME) and minor error (mE) are the numbers in their respective parentheses.

## Discussion

This report delineates a SERS-based protocol of AST for the positive blood-culture isolates from BSI patients. It is composed of four steps: (1) pretreatment of clinical blood-culture samples, (2) drug treatment of the blood-culture isolates in culture medium, (3) acquisition of characteristic SERS spectra of the blood-culture isolates, and (4) identification of antibiotic susceptible profile using characteristic SERS biomarkers. The pretreatment involves a simple sonication of samples in ACK buffer. It allowed *S. aureus* or *E. coli* to be extracted from their respective blood-culture samples successfully for SERS measurement without any observable interference from other constituents in the original blood-culture samples. The SERS biomarkers of the blood-culture isolates after two-hour treatment of antibiotics respond well to their corresponding susceptibility which was verified with standard agar-dilution method. The thus obtained signal ratios of the SERS biomarkers provide a new means to determine antibiotic susceptibility of blood-culture bacterial isolates in a much shorter time period—yielding the rapid SERS-AST method.

The simple and rapid sample pretreatment with ACK buffer followed by sonication is the key additional step that advances the SERS-AST method developed for reference bacterial strains and clinical isolates^31^ to one for clinical blood-culture bacteria. The pretreatment effectively removes various components in blood-culture isolates, which produce substantial interfering SERS signals that overhelm the biomarker signals and render the identification of antibiotic susceptible profile impossible. As shown in Fig. 5, the acquired SERS spectra of the blood-culture samples after the sample pretreatment were almost indistinguishable from that of the corresponding reference strains and pure clinical isolates. This agreement also confirms that the bacteria obtained from various sources (i.e. reference strains, clinical isolates and blood-culture isolates) release the common metabolites of purine derivatives. Among the meddlesome species existing in blood, hemoglobin in red blood cells is particularly the one needed to be removed owing to the strong resonant SERS activity of its heme group (an oxygen-binding iron-coordinated porphyrin^35^). A common solution to this problem is to remove the RBC by differential lysis using ammonium chloride (NH_4_Cl), which is the main constituent of ACK buffer. Since RBC membranes are effectively permeable to NH_4_Cl, cell lysis occurs due to the unbalanced osmotic pressure of their colloid content^45^. Considering its superiority in effectively targeting RBC without destructing bacteria^46^, the choice of ACK buffer appears to be satisfactory. The sequential sonication enhances the lysis of red blood cells through the mechanism of sonoporation^35,36^ and promotes the release of hemoglobin as well as other constituents, which can be effectively removed or diluted by the subsequent washing procedure. The pretreatment step to extract the bacteria from the blood-culture samples of BSI patients has led to recognizable and reproducible bacterial SERS spectra, making identification of antibiotic susceptible profile possible. As a note, Boardman and coworkers^32^ developed a protocol to treat their blood-culture samples. In the protocol, a ‘proprietary lysis buffer’ was used for a lysis treatment of blood cells to remove the possible interference SERS signals from hemoglobin and other constituents in blood. However, they did not adopt the sonication treatment, while we found that such treatment greatly facilitated the lysis process. Finally, we also note that Lorenz and coworkers used another cell-lysis agent (Triton X-100) for the bacterial analysis with normal Raman scattering^47^. They found that the use of additional Pronase E would remove the hemoglobin affixed to the bacteria, owing to its enzymatic hemoglobin digestion, to avoid the interference of hemoglobin in their Raman analysis.

The attainment of the SERS-AST method is established on the prompt response of bacterial metabolomics to stresses induced by antibiotics or other environmental factors. The SERS biomarkers appear to be effective indicators of such metabolic responses. After the 2-h. drug treatment, the samples underwent the washing procedure, before being placed on the SERS substrate for SERS measurements. This step promptly removed the nutrients in the culture media, leaving the live bacteria starved with pure water. Rinas et al. showed that *E. coli* secretes nucleobases (noticeably xanthine) upon entry into the stationary phase (a nutrient-famine stage)^48^. Later, Brauer et al. furthered the study with extended revelation of more intracellular and excellular metabolites^49^. Recently, the exogenous buildup of xanthine and hypoxanthine for *E. coli* in glucose-starved condition was additionally confirmed^50^. Similarly, Liebeke et al. observed adenine secretion from *S. aureus* during glucose starvation^51^. These studies thus lay the foundation for the respective origins of the bacterial SERS spectra observed on *E. coli* and *S. aureus*. To be noted is that the SERS spectrum of *E. coli* is dominated by xanthine and hypoxanthine while that of *S. aureus* by adenine, as observed by Premasiri et al.^33^ and similarly by our group^34^. The agreement of SERS spectra between reference strains, clinical isolates and blood-culture isolates in our study also implicates the common metabolic profiles of purine derivatives of bacteria from these sources. It would be very likely that only very few of all the identified bacterial metabolites (specifically, xanthine and hypoxanthine for *E. coli* and adenine for *S. aureus*) are primarily adsorbed on the SERS-active substrate to produce their respective Raman spectra. This preferential adsorption behavior is reflected by the studies of Premasiri et al.^33^ and our group^34^: the SERS spectra of adenine and xanthine-hypoxanthine mixture are identical to those of *S. aureus* and *E. coli*, respectively, though both bacteria secret more types of molecules. Therefore, the ratio between the SERS biomarker signals ($r_{730}$ for *S. aureus* and $r_{724}$ for *E. coli*) of the samples with and without the treatment of antibiotic reflects the changes of the amount of purine metabolites released per bacterium and of the concentration of live bacteria.

Techniques from the genomics tool box and studies involving transcriptomics, proteomics and metabolomics have helped reveal the behaviors of *E. coli* and *S. aureus* (the prototypical Gram-negative and Gram-positive bacteria, respectively) under differing stressed environments (e.g., nutrient starvation, low/high temperature, high salination, and antibiotic exposure). Particularly, metabolomics quantifies and monitors the variations in bacterial metabolism in response to these environmental conditions, providing relevant information for the development of new antibiotics and the emergence of antibiotic resistance. Belenky and coworkers profiled the metabolome of *E. coli* during the treatment of *β*-lactams, aminoglycodies, and quinolones and found that these treatments induced a similar set of metabolic changes after 30 min.^52^. Most importantly, the concentrations of adenine and guanine were greatly reduced while that of xanthine remains steady in all the three drug-treatment conditions at 30 min. and up to 90 min. These results were further explored by Zampieri et al*.*^53^ with nine antibiotics that represent five mechanisms of actions with better profiling time resolution. These purine metabolites showed similar dynamics for these different antibiotics with respective targets and with distinct dosages, leading them to suggest common metabolic responses to antibiotics owing to single metabolic propagation pathway of perturbations inhibiting essential cellular processes. Although the common adapted metabolic response could counteract the action of antibiotics^53^, it reflects consistent change of purine metabolites upon exposure of all antibiotics and therefore facilitates the development of simple diagnostic markers to the response of susceptible bacteria to differing antibiotics. On the other hand, resistant strains might not bear the same metabolic response because of their distinct metabolic pathways under the antibiotic challenge. Most recently, a study by Collins’s team based on machine learning^54^ has concluded that the purine biosynthesis pathway of susceptible *E. coli* strain is attacked by antibiotics while that of resistant strain is unaffected by it. Subsequently, they further tested the close relationship between the antibiotic lethality and the bacterial metabolic state (using intracellular ATP as a metabolic reporter) on *E. coli*, *S aureus* and *A. baumannii* over nine representative drugs^55^. Their works represent the earliest effort to unravel this mystery and its relevance to the AST based on the prompt response of secreted purine derivatives. In the case of *S. aureus*, Dörries et al*.*^56^ profiled its intra- and extracellular metabolites under the impact of antibiotics with different targets (ciprofloxacin, erythromycin, fosformycin, vancomycin, and ampicillin). Notably, the secreted adenine remained or increased slightly upon the exposure of these antibiotics, reflecting that *S. aureus* and *E. coli* have different metabolic response to antibiotics. Recently, Schelli et al*.*^57^ compared the changes of the metabolic profiles of methicillin-susceptible and resistant *S. aureus* (MSSA and MRSA) under the treatment of ampicillin, kanamycin and norfloxacin. They found that the purine metabolism of MSSA was greatly altered under the treatment of ampicillin and kanamycin and that under the treatment of norfloxacin was slightly dysregulated; in contrast, the changes of the purine metabolism of MRSA upon the exposure of kanamycin and norfloxacin are larger than that under the exposure of ampicillin. Explicitly, the released purines depend on the antibiotics and the bacterial strains (MSSA or MRSA). Although there is no similar study for susceptible and resistant *E. coli*, similar metabolic-response dependence is expected. On the basis of the above studies that many metabolites are promptly released from *E. coli* under antibiotic challenges^52,53^ and of the fact that the SERS spectra of *S. aureus* and *E. coli* are dominated by a few purine derivatives secreted from bacteria under stress^33,34^, one can qualitatively understand why the SERS biomarkers derived from bacterial purine metabolites constitute effective indicators that have been successfully exploited as diagnostic markers for bacterial AST.

To understand the assay principle of SERS-AST quantitatively, we express the SERS spectra $I\left( \nu \right)$ of bacteria as

2 $$I\left( \nu \right) = \mathop \sum \limits_{i = 1}^{N} \hat{I}_{i} \left( \nu \right) \times \theta_{i} ,$$

where $\hat{I}_{i} \left( \nu \right)$ is the SERS spectrum of secreted metabolite *i* at the full coverage of the SERS substrate, $\nu$ is the Raman-shift frequency, and *N* is the number of secreted metabolite types adsorbed on the SERS substrate. Equation (2) is derived from the fact that the SERS activity takes place on the molecules adsorbed on the surface of the SERS substrate^58^. The surface coverage of the metabolite *i*, $\theta_{i}$, depends on the concentration of this metabolite $C_{i}$. This condition is generally true in this study^34^. Therefore, the SERS spectrum of *S. aureus*, $I_{S. a.} \left( \nu \right)$, is simplified as the SERS spectrum of adenine (A), $\hat{I}_{{\text{A}}} \left( \nu \right) \times \theta_{{\text{A}}}$, as adenine molecules predominantly occupy the surface of the SERS substrate. On the other hand, the SERS spectrum of *E. coli*, $I_{E. c.} \left( \nu \right)$, is expressed as $I_{E. c.} \left( \nu \right) = \hat{I}_{{\text{X}}} \left( \nu \right) \times \theta_{{\text{X}}} + \hat{I}_{{{\text{HX}}}} \left( \nu \right) \times \theta_{{{\text{HX}}}}$, where X and HX represent xanthine and hypoxanthine, respectively. This inference given here is derived from the results of the previous study by Premasiri et al.^33^ and with that from our previous work^34^. Note that $C_{i}$ is the concentration of the metabolite *i* released by bacteria in the nutrient-starvation condition after the bacterium-washing step and before the SERS measurement. Namely, if the concentration of the metabolite *i* is low enough such that it is below the metabolite’s solubility in water and the released metabolites do not induce additional metabolic response to the bacteria, the concentration of the metabolite *i* is expressed approximately as

3 $$C_{i}^{0} \approx C_{{\text{B}}}^{0} \times n_{i}^{0} ,$$

where $C_{{\text{B}}}^{0}$ is the concentration of the bacterium and $n_{i}^{0}$ is the number of the metabolite *i* secreted per cell without the antibiotic treatment. After the 2-h. antibiotic treatment, the resultant concentration of the metabolite *i* becomes $C_{i}^{{\text{D}}}$ and can be different from $C_{i}^{0}$, if the drug is effective: $C_{i}^{{\text{D}}} \approx C_{{\text{B}}}^{{\text{D}}} \times n_{i}^{{\text{D}}}$, where $C_{{\text{B}}}^{{\text{D}}}$ and $n_{i}^{{\text{D}}}$ are the respective counterparts. If the drug concentration is much less than MIC or the bacterium strain under test is resistant to the drug, the bacteria may grow and be physiologically active. The ultimate metabolite concentration of the bacteria treated with antibiotic would be comparable to that without the antibiotic treatment. If the drug concentration is higher than MIC, the bacterial amount will be diminished. The ultimate metabolite concentration would decline drastically. Following the consideration in Eq. (3), the signal ratio of the SERS biomarker at the Raman shift of $\nu_{{{\text{bm}}}}$ that is characteristic of some specific metabolite corresponding to $\nu_{{{\text{bm}}}}$, $r_{{\nu_{{{\text{bm}}}} }}$, reflects the change of the concentration of that metabolite upon the exposure of antibiotic and can be expressed as

4 $$r_{{\nu_{{{\text{bm}}}} }} = S_{{\nu_{{{\text{bm}}}} }}^{{\text{D}}} /S_{{\nu_{{{\text{bm}}}} }}^{0} \approx \theta_{{\nu_{{{\text{bm}}}} }}^{{\text{D}}} /\theta_{{\nu_{{{\text{bm}}}} }}^{0} ,$$

where $S_{{\nu_{{{\text{bm}}}} }}^{{\text{D}}}$ and $S_{{\nu_{{{\text{bm}}}} }}^{0}$ are the bacterial SERS signals at $\nu_{{{\text{bm}}}}$ with and without the antibiotic treatment, respectively. $\theta_{{\nu_{{{\text{bm}}}} }}^{{\text{D}}}$ and $\theta_{{\nu_{{{\text{bm}}}} }}^{0}$ are the corresponding surface coverages of the biomarker metabolite. Therefore, after the treatment of antibiotic for 2 h., two possible scenarios would take place for the treated bacteria: (1) if the drug concentration is much less than MIC or the bacterium under test is resistant to the drug, the bacterial concentration with the antibiotic treatment, $C_{{\text{B}}}^{{\text{D}}}$, is comparable to the bacterial concentration without the antibiotic treatment, $C_{{\text{B}}}^{0}$,—namely $C_{i}^{{\text{D}}} \approx C_{i}^{0}$, resulting in $\theta_{i}^{{\text{D}}} \approx \theta_{i}^{0}$ and thus $r_{{\nu_{{{\text{bm}}}} }} \approx 1$; (2) if the drug concentration is higher than MIC and the bacterium is susceptible to the drug, $C_{{\text{B}}}^{{\text{D}}}$ is much smaller than $C_{{\text{B}}}^{0}$—namely $C_{{\text{B}}}^{0} \gg C_{{\text{B}}}^{{\text{D}}} \approx 0$, resulting in $\theta_{i}^{0} \gg \theta_{i}^{{\text{D}}} \approx 0$ and accordingly $1 \gg r_{{\nu_{{{\text{bm}}}} }} \approx 0$. Besides, as the drug concentration is comparable to MIC (or in other words, the drug concentration is sub-lethal), $C_{{\text{B}}}^{{\text{D}}}$ can be only slightly smaller than $C_{{\text{B}}}^{0}$. Some blood-culture isolates may respond differently to antibiotic such that the biomarker metabolite released after the antibiotic treatment is higher than that without the treatment the bacteria: $n_{i}^{{\text{D}}} > n_{i}^{0}$. This fact might add some uncertainty in the outcome of $r_{{\nu_{{{\text{bm}}}} }}$ in the sub-lethal drug concentration range, probably yielding some disagreement in the obtained MIC based on the SERS-AST results. However, the very high sensitivity and specificity of the SERS-AST method shown in this proof-of-concept study suggest the inconsequential effect of such uncertainty.

The success of the SERS-AST method relies on our previous efforts in developing the SERS detection platform: (1) the development of uniform and reliable SERS-active substrate based on 2-dimension Ag nanoparticle array embedded in nanochannels of anodic alumina^44^, (2) the experimental and theoretical investigations of its anomalous optical propensity^59–61^, and (3) the fundamental understanding of SERS mechanisms^61–64^. There are several points that are relevant to the SERS-AST method and worthy of special attention. First, the SERS spectrum of bare substrate shows featureless continuum background, giving minimal interference to the bacterial SERS signal. Second, the SERS signal of adenine—used in quality-control test of the substrates—varies for about 10% over centimeters of the substrate and the normalized SERS spectra from different sites within individual sample spots are virtually indistinguishable (less than 1% of variation). Third, the signal-to-background ratio (S/B) also shows similarly small variation and is higher than unity for quantitative analysis. The highly reproducibility and comparably large S/B value of the SERS signal therefore have enabled the quantitative comparison of the ratios of the SERS biomarker signals ($r_{730}$ or $r_{724}$) between the antibiotic-treated sample and the control sample nearby (Figs. 6 and 7) to determine the antibiotic susceptibility and MIC.

Finally, the SERS-AST protocol is carried out on positive blood-culture samples in this study—namely, the AST is performed after incubation of blood samples phlebotomized from patients. On the other hand, there have been new molecular diagnostic methods developed to tackle whole blood specimens—the blood samples drawn from patients—without elongated blood culture, as the efforts to shorten the turnaround time for the AST results^65^. They can be mainly categorized into two types: nucleic acid amplification technologies (NAAT) and amplification-free technology. The first type works based on the PCR scheme to increase the quantity of the matched DNA or RNA targets, while the second type deploys specific labeling and/or microscopic technology to detect the targeted pathogens in single-cell level. These technologies aim to obtain the bacterial identification and the AST result without the blood culture procedure. Several challenges however appear in the implementation of these technologies^66^. First, the NAATs detect circulating microbial DNA or RNA in blood which may not correspond to viable bacteria after blood culture. Second, the co-present cellular materials and anticoagulants in whole blood may inhibit PCR reactions. Third, the whole blood samples from healthy donors and patients after successful treatment may still contain bacterial DNA/RNA, leading to false positive results. Fourth, different phenotypes exist even for isogenetic cells owing to the stochasticity in gene expression induced by fluctuated molecular interaction, local environmental differences—including both intrinsic and extrinsic biological noises^67^, and asynchrony in cell division and ageing^68,69^, thus setting a fundamental limit on microbiological diagnosis based on few cells. These challenges do impose a doubt whether these culture-free technologies can be implemented in actual clinical settings. Blood culture in fact not only amplifies the quantity of bacteria in the drawn blood samples, but also ensures their viability—therefore, among the bacteria with variant physiological conditions due to medical treatments (including antibiotics), it selects the one that is most evolutionally fit the culture environment. Accordingly, blood culture is similarly needed for almost all the existing and developing AST methods, even though some technologies do hold single-cell detectivity. The challenge is whether it is possible to culture the bacteria in a short period such that the uniformity in their physiological conditions is reached and the cell quantity is large enough so that the statistically meaningful AST results can be obtained. With this consideration, it is very possible that the period of blood culture can be shortened for the detection limit of bacteria of 10^5^~10^6^ CFU/ml in the SERS-AST protocol.

## Conclusions

In summary, this is the first proof-of-concept study that demonstrated the successful application of SERS technology for clinical AST from positive blood-culture isolates of *S. aureus* and *E. coli*. An efficient sample pretreatment protocol was developed to recover bacterial isolates that transpire SERS spectra almost identical to those of their corresponding reference strains. The antimicrobial activity of tested drugs was accurately indicated by signal ratios of SERS biomarkers with high specificity and sensitivity. The rapid and reliable AST results yielded by SERS would be of vital importance not only to guide the early antibiotic therapy but also to reduce the emergence of antimicrobial-resistant strains. The inspiring results highlight that, from sample to identification, the SERS technology has revolutionized the way bacteremic samples are screened for pathogens as well as their resistogram. Further researches involving more bacterial species and antibiotics with clinical significance are needed to advance the prototype of SERS-AST into the current workflow of microbiological examination for blood stream infections.

### Ethical statement

This study protocol was approved by the research ethics committee of NTUH (case number 201107031RC) and conducted according to Good Clinical Practice guidelines and the recommendations of the Declaration of Helsinki. Written informed consent was obtained from each patient who decided to participate before sample acquirement.

## Supplementary information

Supplementary information
