# Supplementary material for: Rapid antibiotic susceptibility testing of bacteria from patients’ blood via assaying bacterial metabolic response with surface-enhanced Raman spectroscopy
Source: Sci Rep. 2020 Jul 27;10:12538. doi: 10.1038/s41598-020-68855-w (PMC7385103; doi:10.1038/s41598-020-68855-w)
Supplement: Supplementary file 1 — Supplementary information [file 41598_2020_68855_MOESM1_ESM.docx]

Supporting Information

**Rapid Antibiotic Susceptibility Testing of Bacteria from Patients’ Blood via Assaying Bacterial Metabolic Response with Surface-Enhanced Raman Spectroscopy**

Yin-Yi Han^1,2*^, Yi-Chun Lin^3^, Wei-Chih Cheng^3^, Yu-Tzu Lin^4^, Lee-Jene Teng^4^, Juen-Kai Wang^3,5,6*^ & Yuh-Lin Wang^3,7*^

^1^ Department of Anesthesia, National Taiwan University Hospital, Taipei, Taiwan

^2^ Department of Traumatology, National Taiwan University Hospital, Taipei, Taiwan

^3^Institute of Atomic and Molecular Sciences, Academia Sinica, Taipei, Taiwan

^4^Department of Clinical Laboratory Sciences and Medical Biotechnology, National Taiwan University

College of Medicine, Taipei, Taiwan

^5^Center for Condensed Matter Sciences, National Taiwan University, Taipei, Taiwan

^6^Center of Atomic Initiative for New Materials, National Taiwan University, Taipei, Taiwan

^7^Department of Physics, National Taiwan University, Taipei, Taiwan.

*Corresponding authors: noviahan@gmail.com, jkwang@ntu.edu.tw and ylwang@pub.iams.sinica.edu.tw

**Table S1.** Ratios between the SERS marker signals of blood-culture isolates with and without corresponding antibiotic treatment. $r_{730}$ and $r_{724}$ are the ratios at 730 and 724 cm^-1^, respectively.

| Causative pathogen | *S. aureus* | | | | | *E. coli* | | | | |
| --- | --- | --- | --- | --- | --- | --- | --- | --- | --- | --- |
| Signal ratio of biomarker | $r_{730}$ | | | | | $r_{724}$ | | | | |
| Drug concentration  (*μ*g/ml) | Case no. | 0.5 | 1 | 2 | 4 | Case no. | 0.5 | 1 | 2 | 4 |
| Susceptible blood isolate | GP005 | 0.55 | 0.15 | 0.14 | 0.12 | GN005 | 0.07 | 0.10 | 0.07 | 0.07 |
|  | GP006 | 0.43 | 0.25 | 0.19 | 0.16 | GN007 | 0.12 | 0.06 | 0.08 | 0.02 |
|  | GP007 | 0.14 | 0.08 | 0.10 | 0.12 | GN008 | 0.14 | 0.13 | 0.10 | 0.11 |
|  | GP014 | 0.13 | 0.14 | 0.20 | 0.07 | GN009 | 0.87 | 0.15 | 0.05 | 0.10 |
|  | GP016 | 0.03 | 0.05 | 0.05 | 0.04 | GN010 | 0.26 | 0.17 | 0.09 | 0.09 |
|  | GP017 | 1.24 | 0.68 | 0.27 | 0.20 | GN014 | 0.50 | 0.25 | 0.20 | 0.34 |
|  | GP019 | 2.45 | 1.06 | 0.55 | 0.48 | GN020 | 0.14 | 0.12 | 0.11 | 0.09 |
|  | GP020 | 0.45 | 0.17 | 0.09 | 0.08 | GN022 | 0.41 | 0.10 | 0.07 | 0.02 |
|  | GP021 | 1.17 | 0.50 | 0.27 | 0.20 | GN027 | 0.27 | 0.15 | 0.14 | 0.12 |
|  | GP022 | 0.20 | 0.28 | 0.13 | 0.11 | GN028 | 0.45 | 0.35 | 0.08 | 0.05 |
|  | GP023 | 0.45 | 0.42 | 0.14 | 0.21 | GN029 | 0.90 | 0.39 | 0.08 | 0.09 |
|  | GP024 | 0.54 | 0.21 | 0.15 | 0.18 | GN030 | 0.31 | 0.48 | 0.16 | 0.11 |
|  | GP025 | 0.38 | 0.25 | 0.20 | 0.19 | GN032 | 0.20 | 0.25 | 0.16 | 0.14 |
|  | GP028 | 0.47 | 0.21 | 0.27 | 0.20 | GN034 | 0.25 | 0.13 | 0.04 | 0.13 |
|  | GP030 | 0.22 | 0.16 | 0.13 | 0.14 | GN038 | 0.25 | 0.10 | 0.08 | 0.06 |
|  | GP032 | 0.34 | 0.12 | 0.14 | 0.14 | GN040 | 1.14 | 1.21 | 0.50 | 0.27 |
|  | GP040 | 0.31 | 0.09 | 0.09 | 0.07 | GN041 | 0.30 | 0.24 | 0.20 | 0.19 |
|  |  |  | | | | GN042 | 0.15 | 0.12 | 0.09 | 0.10 |
|  |  |  |  |  |  | GN043 | 0.87 | 0.30 | 0.19 | 0.11 |
|  |  |  |  |  |  | GN046 | 0.68 | 0.12 | 0.08 | 0.08 |
|  |  |  |  |  |  | GN047 | 1.07 | 0.63 | 0.33 | 0.04 |
|  |  |  |  |  |  | GN048 | 0.99 | 0.22 | 0.27 | 0.08 |
|  |  |  |  |  |  | GN049 | 0.19 | 0.08 | 0.06 | 0.03 |
|  |  |  |  |  |  | GN050 | 0.49 | 0.50 | 0.23 | 0.10 |
|  |  |  |  |  |  | GN054 | 0.35 | 0.19 | 0.09 | 0.12 |
|  |  |  |  |  |  | GN055 | 0.29 | 0.25 | 0.12 | 0.08 |
|  |  |  |  |  |  | GN056 | 0.89 | 0.64 | 0.11 | 0.07 |
|  |  |  |  |  |  | GN060 | 0.12 | 0.05 | 0.04 | 0.10 |
|  |  |  |  |  |  | GN063 | 0.17 | 0.20 | 0.18 | 0.08 |
|  |  |  |  |  |  | GN065 | 0.43 | 0.40 | 0.14 | 0.07 |
| Resistant blood isolate | GP008 | 0.84 | 0.84 | 0.91 | 0.80 | GN006 | 0.64 | 0.88 | 0.85 | 0.82 |
|  | GP009 | 0.88 | 0.87 | 1.17 | 0.86 | GN012 | 0.97 | 0.86 | 1.27 | 1.25 |
|  | GP010 | 0.97 | 1.11 | 0.91 | 0.92 | GN013 | 1.04 | 0.98 | 1.07 | 0.90 |
|  | GP011 | 0.94 | 0.81 | 1.20 | 0.94 | GN015 | 0.87 | 0.78 | 0.78 | 1.04 |
|  | GP012 | 1.36 | 1.16 | 1.22 | 0.86 | GN018 | 1.19 | 0.84 | 0.91 | 0.48 |
|  | GP013 | 1.47 | 0.95 | 0.96 | 0.90 | GN019 | 0.69 | 1.87 | 1.48 | 0.17 |
|  | GP026 | 0.70 | 1.14 | 1.44 | 1.17 | GN024 | 0.82 | 0.88 | 1.04 | 1.07 |
|  | GP027 | 0.94 | 0.62 | 0.84 | 0.56 | GN033 | 0.60 | 1.74 | 1.74 | 1.16 |
|  | GP031 | 0.38 | 0.25 | 0.20 | 0.09 | GN035 | 0.67 | 0.62 | 0.82 | 0.99 |
|  | GP033 | 0.89 | 0.73 | 0.40 | 0.23 | GN037 | 1.44 | 1.08 | 1.21 | 0.75 |
|  | GP034 | 0.84 | 0.76 | 0.44 | 0.18 | GN039 | 1.06 | 0.78 | 0.84 | 0.52 |
|  | GP035 | 1.25 | 1.02 | 0.89 | 1.27 | GN044 | 0.88 | 1.26 | 1.06 | 1.47 |
|  | GP037 | 0.63 | 0.56 | 0.90 | 1.02 | GN045 | 2.14 | 2.06 | 2.47 | 1.15 |
|  | GP038 | 1.05 | 1.03 | 1.03 | 1.44 | GN059 | 1.11 | 2.30 | 0.66 | 2.01 |
|  | GP039 | 0.60 | 0.51 | 0.86 | 0.91 | GN061 | 0.90 | 0.86 | 0.62 | 0.39 |


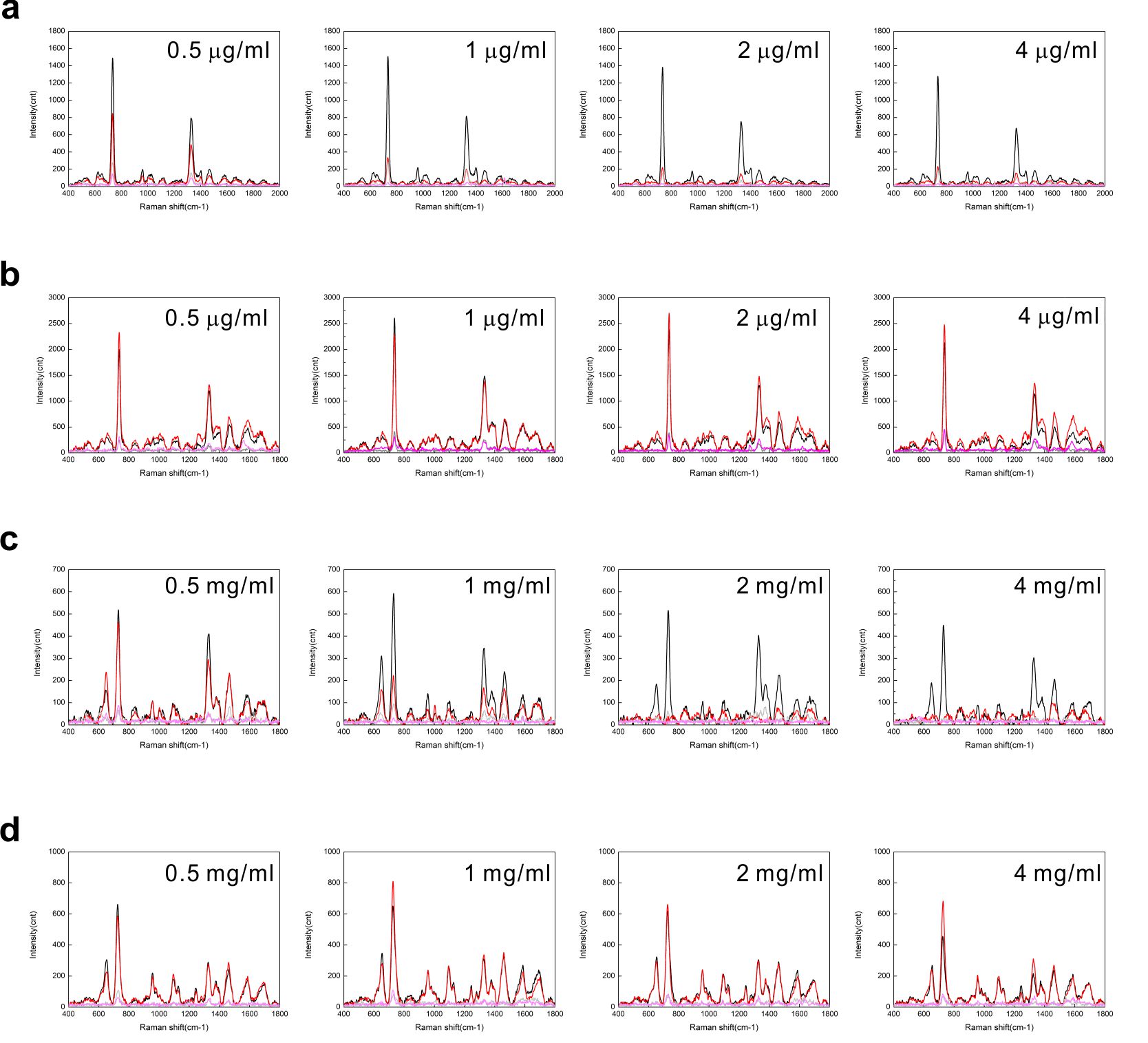


**Figure S1.** SERS spectra (red lines) of blood-cultured **a** susceptible and **b** resistant *S. aureus* treated with oxacillin concentrations of 0.5, 1, 2 and 4 mg/ml; SERS spectra of blood-cultured **c** susceptible and **d** resistant *E. coli* treated with cefotaxime concentrations of 0.5, 1, 2 and 4 *μ*g/ml. The corresponding SERS spectra (black lines) without antibiotic treatment are shown for comparison. Light red and gray curves represent their respective standard deviations.
